# Development of an Acoustic System for UAV Detection †

**DOI:** 10.3390/s20174870

**Published:** 2020-08-28

**Authors:** Cătălin Dumitrescu, Marius Minea, Ilona Mădălina Costea, Ionut Cosmin Chiva, Augustin Semenescu

**Affiliations:** 1 Department Telematics and Electronics for Transports, University “Politehnica” of Bucharest, 060042 Bucharest, Romania; marius.minea@upb.ro (M.M.); ilona.costea@upb.ro (I.M.C.); cosmin377@gmail.com (I.C.C.); 2 Department Engineering and Management for Transports, University “Politehnica” of Bucharest, 060042 Bucharest, Romania; augustin.semenescu@upb.ro

**Keywords:** microphone array, concurrent neural networks, sensors, microsystems, drone detection

## Abstract

The purpose of this paper is to investigate the possibility of developing and using an intelligent, flexible, and reliable acoustic system, designed to discover, locate, and transmit the position of unmanned aerial vehicles (UAVs). Such an application is very useful for monitoring sensitive areas and land territories subject to privacy. The software functional components of the proposed detection and location algorithm were developed employing acoustic signal analysis and concurrent neural networks (CoNNs). An analysis of the detection and tracking performance for remotely piloted aircraft systems (RPASs), measured with a dedicated spiral microphone array with MEMS microphones, was also performed. The detection and tracking algorithms were implemented based on spectrograms decomposition and adaptive filters. In this research, spectrograms with Cohen class decomposition, log-Mel spectrograms, harmonic-percussive source separation and raw audio waveforms of the audio sample, collected from the spiral microphone array—as an input to the Concurrent Neural Networks were used, in order to determine and classify the number of detected drones in the perimeter of interest.

## 1. Introduction

In recent years, the use of small drones has increased dramatically. Illegal activity with these UAVs has also increased, or at least became more evident than before. Recently, it has been reported that such vehicles have been employed to transport drugs across borders, to transport smugglers to prisons, to breach the security perimeter of airports and to create aerial images of senzitive facilities. To help protect against these activities, a drone detection product could warn of a security breach in due time to take action. This article tries to answer the following questions:

(1) Is it possible to build an audio detection, recognition, and classification system able to detect the presence of several drones in the environment, with relatively cheap commercial equipment (COTS)?
(2) Assuming that it can function as a prototype, what challenges could be raised when scaling the prototype for practical use?
(3) Are the techniques used in the development of the prototype drone detection, recognition, and classification superior in performance to existing commercial systems?

The questions will be approached in the context of a comparison between the performance of systems using concurrent neural networks and the algorithm proposed by the authors. The proposed solution employs for the acoustic drone detector competing neural networks with spectrogram variants both in frequency and psychoacoustic scales, and increased performance for neural network architectures.

Two concepts are investigated in this work: (i) the way that a concept of competition in a collection of neural networks can be implemented, and (ii) how different input data can influence the performance of the recognition process in some types of neural networks.

The subject of this article is in the form recognition domain, that offers a very broad field of research. Recognition of acoustic signatures is a challenging task, grouping a variety of issues, which include the recognition of isolated characteristic frequencies and identification of unmanned aerial vehicles, based on their acoustic signatures. Neural networks represent a tool that has proven its effectiveness in solving a wide range of applications, including automated speech recognition. Most neural models approach form recognition as a unitary, global problem, without distinguishing between different input intakes. It is a known fact that the performance of neural networks may be improved via modularity and by applying the “divide et impera” principle. In this paper, the identification and classification of UAVs is performed by the means of two neural networks: the self- organizing map (SOM) and the concurrent neural network (CoNN). The newly introduced CoNN model combines supervised and unsupervised learning paradigms and provides a solution to the first problem. A process of competition is then employed in a collection of neural networks that are independently trained to solve different sub-problems. This process is accomplished by identifying the neural network which provides the best response. As experimental results demonstrate, a higher accuracy may be obtained when employing this proposed algorithm, compared to those employed in non-competitive cases.

Several original recognition models have been tested and the theoretical developments and experimental results demonstrate their viability. The obtained databases are diverse, being both standard collections for different types of UAVs’ soundings and sets made specifically for the experiments in this paper, containing acoustic signatures of proprietary drones. Based on the tests performed on some models and standard form recognition data sets, it can be illustrated that these may be also used in contexts other than the recognition of acoustic signals generated by drones. In order to reduce the complexity of recognition through a single neural network of the entire collection of isolated acoustic frequency of all drones, a solution of a modular neural network, consisting of neural networks specialized on subproblems of the initial problem has been chosen. The concurrent neural networks classification has been introduced as a collection of low-volume neural networks working in parallel, where the classification is made according to the rule where the winner takes all. The training of competing neural networks starts from the assumption that each module is trained with its own data set. The system is made up of neural networks with various architectures. Multi-layered perceptron types, time-lagged and self-mapping neural network types have been used for this particular case, but other variants may also be employed. The recognition scheme consists of a collection of modules trained on a subproblem and a module that selects the best answer. The training and recognition algorithms implement these two techniques that are custom for multilayer perceptron (MLP), time delayed neural networks (TDNN) and self-organizing maps (SOM). MLP-CoNN and TDNN-CoNN use supervised trained modules and training instruction sets contain both positive and negative examples. In contrast, SOM-CoNN consists of modules that are trained by an unsupervised algorithm and the data consist only of positive examples.

The remaining of this article is organized as follows: Section 2 presents a selective study on similar scientific works (Related Work), Section 3, the Problem Definition and the Proposed Solution, Section 4, Employing CoNNs in the UAV Recognition Process, Section 5 our Experimental Results and Section 6, the Discussion and Conclusions.

## 2. Related Work

At present the use of UAVs for different tasks has become very popular. Ranging from the military domain, where drones, flying wings or other types of UAVs have gain their precise role, the civil domain has also gathered a series of services that make use of these versatile devices: mail delivery, aerial photogrammetry and measurements, aerial surveillance, and others. Recently, with the rapid expansion of the COVID-19 illness, drones have been employed for aerial detection, spraying disinfectants, surveillance, public announcements, delivery of medical supplies, provision of communication services, and so on [1]. The palette of services offered and the future possibilities are extremely wide. Due to the intensification of these devices’ usage, one problem that is arising is the compliance with security and privacy regulations, both from the point of view of the UAV users, and from the point of view of the overflown territory. There are areas where unauthorized flying of UAVs might involve serious breaches of security and safety, or be even disastrous: airports, military facilities, hospitals, prisons, border lines, nuclear power plants, flammable materials depots, oil refineries and the list could continue. The most significant example is where UAVs are used to remotely capture video footage in areas within a property where privacy is expected. Therefore, it is considered crucial that such areas and facilities need 24/7 automated surveillance for detecting unauthorized fly of such objects over sensitive areas. During the past years since UAVs have been invented, different solutions for their detection have been tested or employed: reception and analysis of emitted radio signals (emitted by drones or their associated equipment), acoustic patterns analysis, video processing, IR imaging, radar, lidar, and so on. Each technology usually has its own advantages and drawbacks. The scientific literature in this research area is relatively rich and several authors present their results. In [2], Jeon et al. perform an investigation of a deep neural network possibility to detect commercial UAVs in environment by analyzing their sound characteristics. The purpose of their experiments is to detect potentially malicious or terrorist aerial vehicles. The authors make use of Gaussian mixture model (GMM), convolutional neural network (CNN) and recurrent neural network (RNN) to recognize commercial UAVs flying in a typical environment. They gathered an F-score recognition of 0.8009 when employing the RNN methodology, with 240 ms of signal input and short processing time. The authors also declared that the most difficult challenge of this type of work was the system training in the presence of environmental noise, in specific restricted areas, where flying of drones was not allowed. However, artificial intelligence trends to be used more and more in this area of research.

In [3], the authors present the results of a cost-effective RF-based method for detecting UAVs. They propose two different approaches: (i) active tracking: the detection system sends a radio signal and then listens for its reflected component (RADAR principle). (ii) passive tracking: the system receives, extracts, and then analyzes the acquired radio signal. The paper also proposes two methods, active and passive. The active method concerns the observations on the reflected radio signals, while the passive by analyzing the communication between the UAV and its controller. A combined solution for detecting both flight-enabled drones and land-mobile drones is presented in Patent [4], where a network of different types of sensors is deployed around the surveilled property. The signals provided by the sensors network is analyzed in the native surveillance application, resident in the central computer of the network. In patent [5], the inventors present a system, method and apparatus for drone detection that include a microphone, a sound card, and a computer. A wide category of sensors used for drone detection is represented by the electro-optical, infrared (EO/IR) type, that are frequently capturing environmental imaging for detecting unauthorized flying objects. Distinguishing from the normal daylight imaging sensors, these are capable of detecting targets in low lighting. However, in image recognition technology, the environmental objects such as mountains, trees, buildings, etc. may be backgrounding the subject, i.e., the drones to be detected. Even more, 3D sensors can be used to overcome the limitations of EO/IR sensors in UAV detection. The authors of [6] developed a sensor able to detect high-resolution points within 0.5° × 0.5° by scanning the high-resolution laser pulses at high speed in AZ and EL directions, where intersection angle of the vertical direction (EL direction) is 0.003°, and the intersection angle of horizontal (AZ direction) is 0.011°. Potentially harmful cyber and physical threats that may arise from unauthorized flying of UAVs over forbidden zones is analyzed by other researchers [7], along with reviewing various UAV detection techniques based on ambient radio frequency signals (emitted from drones), radars, acoustic sensors, and computer vision techniques for detection of malicious UAVs.

In s similar work [8], the detection and tracking of multiple UAVs flying at low altitude is performed with the help of a heterogeneous sensor network consisting of acoustic antennas, small frequency modulated continuous wave (FMCW) radar systems and optical sensors. The researchers applied acoustics, radar and lidar to monitor a wide azimuthal area (360°) and to simultaneously track multiple UAVs, and optical sensors for sequential identification with a very narrow field of view. In [9] the team presents an experimental system dedicated for the detection and tracking of small aerial targets such as unmanned aerial vehicles (UAVs) in particular small drones (multi-rotors). A system for acoustic detection and tracking of small objects in movement, such as UAVs or terrestrial robots, using acoustic cameras is introduced in [10]. In their work, the authors deal with the problem of tracking drones in outdoor scenes, scanned by a lidar sensor placed on the ground level. For detecting UAVs the researchers employ a convolutional neural network approach. Afterwards, Kalman filtering algorithms are used as a cross-correlation filtering, then a 3D model is built for determining the velocity of the tracked object. Other technologies involved in unauthorized flying of drones over restricted areas include passive bistatic radar (PBR) employing a multichannel system [11].

In what concerns the usage of deep neural networks in this field of activity, Aker and Kalkan [12] present a solution using an end-to-end object detection model based on convolutional neural networks employed for drone detection. The authors’ solution is based on a single shot object detection model, YOLOv2 [13], which is the follow-up study of Yolo W. For a better selection of UAVs from the background, the model is trained to separate these flying objects from birds. In the conclusion section, the authors state that by using this method drones can be detected and distinguished from birds using an object detection model based on a CNN. Further on, Liu et al. [14] employ an even more complex system for drone detection, composed from a modular camera array system with audio assistance, which consists of several high-definition cameras and multiple microphones, with the purpose to monitor UAVs. In the same area of technologies, Popovic et al. employ a multi-camera sensor design acquiring near-infrared (NIR) spectrum for detecting mini-UAVs in a typical rural country environment. They notice that the detection process needs detailed pixel analysis between two consecutive frames [15]. Similarly, Anwar et al. perform drone detection by extracting the required features from ADr sound, Mel frequency cepstral coefficients (MFCC), and implementing linear predictive cepstral coefficients (LPCC). Classification is performed after the feature extraction, and support vector machines (SVM) with various kernels are also used for improving the classification of the received sound waves [16]. Supplementary, the authors state that *“… the experimental results verify that SVM cubic kernel with MFCC outperform LPCC method by achieving around 96.7% accuracy for ADr detection*”. Moreover, the results verified that the proposed ML scheme has more than 17% detection accuracy, compared with correlation-based drone sound detection scheme that ignores ML prediction. A study on the cheap radiofrequency techniques for detecting drones is presented by Nguyen et al. [17], where they focus on autonomously detection and characterization of unauthorized drones by radio frequency wireless signals, using two combined methods: sending a radiofrequency signal and analyzing its reflection and passive listening of radio signals, process subjected to a second filtration analysis.

An even more complex solution for drone detection using radio waves is presented by Nuss et al. in [18], where the authors employ a system setup based on MIMO OFDM radar that can be used for detection and tracking of UAVs on wider areas. Keeping the research in the same field, the authors of [19] present an overview on passive drone detection with a software defined radio (SDR), using two scenarios. The authors state that “*operation of a non-LoS environment can pose a serious challenge for both passive methods*”. It has been shown that the drone flight altitude may play a significant role in determining the Rician factor and LoS probability, which in turn affects the received SNR. Several other approaches are presented in similar work [20,21].

In what concerns the acoustic signature recognition, the scientific literature is comparatively rich. Bernadini et al. obtained a resulting accuracy of the drone recognition of 98.3% [22]. Yang et al. also propose an UAV detection system with multiple acoustic nodes using machine learning models, with an empirically optimized configuration of the nodes for deployment. Features including Mel-frequency cepstral coefficients (MFCC) and short-time Fourier transform (STFT) were used by these researchers for training. Support vector machines (SVM) and convolutional neural networks (CNN) were trained with the data collected in person. The purpose was to determine the ability of this setup to track trajectories of flying drones [23]. In noisy environments, sound signature of UAVs is more difficult to recognize. Moreover, there are different environments with specific background soundings. Lin Shi et al. deal with this challenge and present an approach to recognize drones via sounds emitted by their propellers. In their paper, the authors declare that experimental results validate the feasibility and effectiveness of their proposed method for UAV detection based on sound signature recognition [24]. Similar work is described in papers [25] and [26]. Finally, it can be concluded that this research field is very active and there are several issues that haven’t been yet fully addressed, such as separation of the UAV from environment (birds, obstructing trees, background mountains, etc.), issue depending very much on the technology chosen for drone detection. However, one approach proves its reliability—that is the usage of multisensory constructions, where weaknesses of some technologies can be compensated by others. Therefore, we consider that employing a multisensory approach has more chances of success than using a single technology.

## 3. Proposed Solution

Classification of environmental sound events is a sub-field of computational analysis of auditory scenes, which focuses on the development of intelligent detection, recognition, and classification systems. Detecting the acoustic fingerprints of drones is a difficult task because the specific acoustic signals are masked by the noises of the detection environment (wind, rain, waves, sound propagation in the open field/urban areas). Unlike naturally occurring sounds, drones have distinctive sound characteristics. Taking advantage of this aspect, the first part of the article focuses on building an audio detection, recognition, and classification system for the simultaneous detection of several drones in the scene.

### 3.1. Proposed Framework and System Architecture

As presented in the initial part of this work, the main task of the proposed system is to detect unauthorized flying of UAVs over restricted areas, by locating these vehicles and tracking them. The difficulty of the process resides in the environmental noise, and the visibility at the moment of detection.

**Figure 1:**
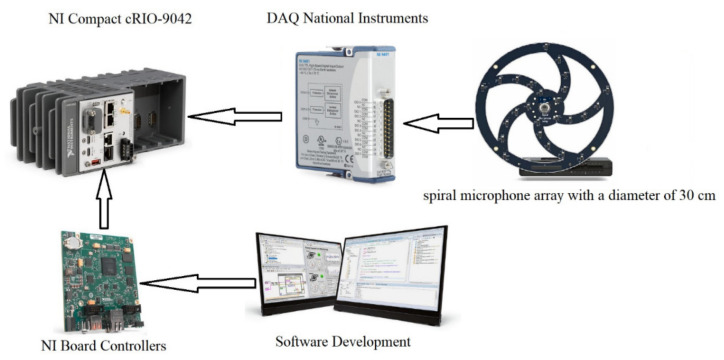
Proposed System’s Hardware Architecture.

**Figure 2:**
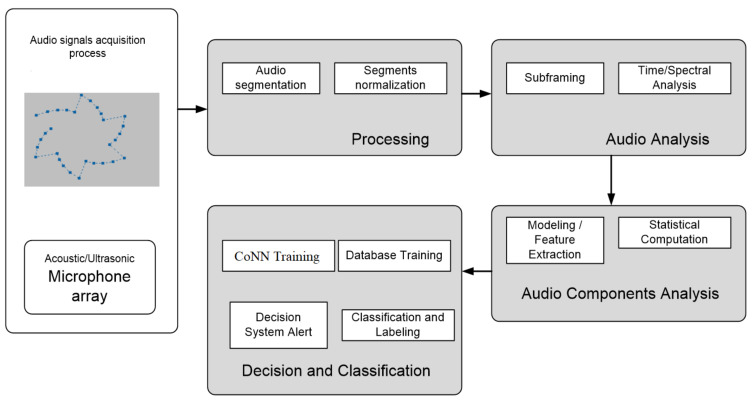
Proposed System’s Software Architecture.

Different types of microphones and a specific arrangement is used for improving the performance of the acoustic detection component. Thus, the system employed for detection, recognition and automatic classification of drones using the acoustic fingerprint is composed of a hardware—software assembly as shown in Figure 1 and Figure 2.

The first functional component to be addressed is the sensing block, composed of an area of spiral-type microphones with MEMS, in the acoustic fields, with a spiral arrangement, shown in Figure 2. The microphone area is composed of 30 spiral-shaped MEMS digital microphones, so as to achieve adaptive multi-channel type weights with variable pitch. The following components have been employed for the microphone array: Knowles (Knowles Electronics, LLC, Itasca, IL, USA) MEMS microphones with good acoustic response types (typically 20 Hz to >20 KHz +/− 2 dB frequency ratings). The system allows the detection of the presence of the acoustic signal of reduced complexity. For improving the quality of the received signal, adaptive methods to cancel the acoustic reaction, as well as adaptive methods to reduce the acoustic noise were also used.

The hardware acoustic system (show in Figure 1) is developed on a National Instruments (NI, Austin, TX, USA) configuration consisting of a NI Compact cRIO-9042 (NI, Austin, TX, USA), NI board controller (Core i7 processor/3.1 GHz, Intel, 8 GB DDR3, Kintex—7 XC7K2160T FPGA, Xilinx), (NI, Austin, TX, USA) and DAQ-9401 acquisition cards (DAQ - NI, Austin, TX, USA). The board controller is a high-performance embedded computer. The run—time software stack includes LabVIEW Real-Time Module and FPGA Module. For the protection of the restricted area, the developed acoustic system was configured in a network composed of at least eight microphone array modules, arranged on the perimeter of the protected area. To increase the detection efficiency, the number of microphone array may also be increased, and the network of acoustic sensors can be configured both linearly and in depth, thus forming a safety zone around the protected area.

Performing acoustic measurements highlights the presence of a tonal component at frequencies of 200–5000 Hz (small and medium drones—multicopter) and in the frequency range 200–10,000 Hz (medium and large drones—multicopter), which is the typical sound emission of UAV in the operation phase of flight. For medium and large multicopter drones the harmonics of the frequencies characteristic are also found over 10 kHz (16–24 kHz). The identification of this frequency is a sign of the presence of a UAV in the environment.

**Figure 3:**
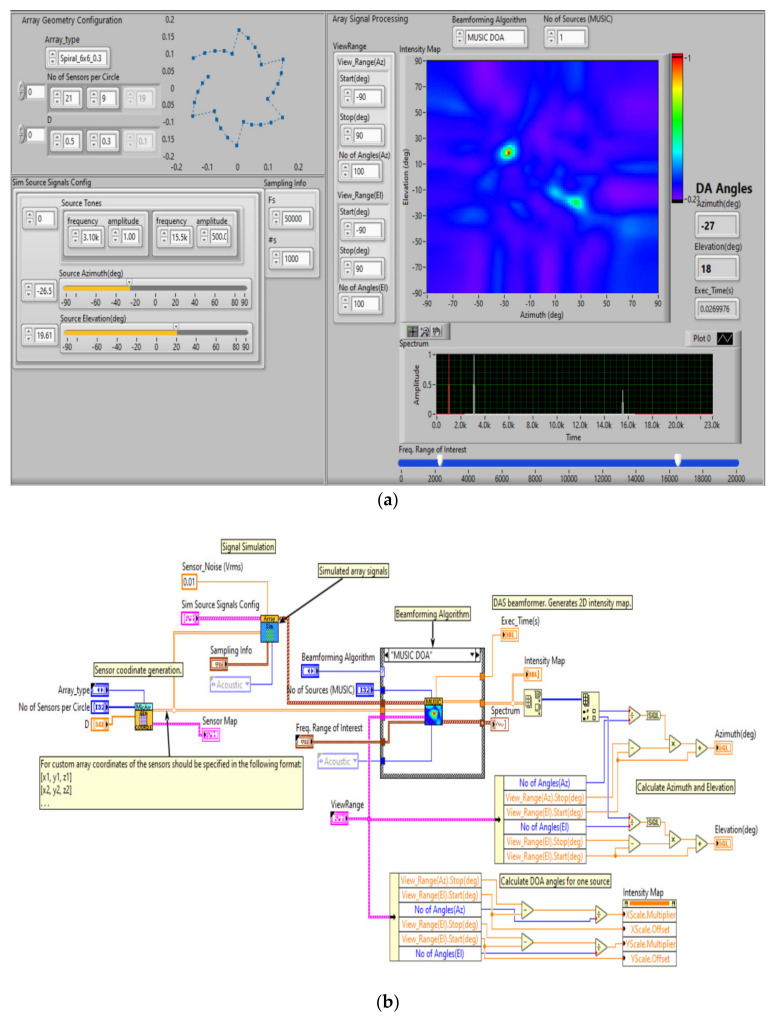
Simulation Spiral Microphone Array. GUI beamforming analysis using acoustic frequency characteristic of UAVs (**a**) and software implementation (**b**).

In Figure 3 is presented the spiral microphone array simulation along with the beamforming analysis using multiple signal classification & direction of arrival (MUSIC DOA). DOA denotes the direction from which typically a propagation wave arrives at a point where a set of sensors are placed.

The image in the right section shows the energetic detection of the acoustic signal generated by the drone’s engines and rotors, detecting the location position (azimuth and elevation), for the two acoustic frequencies characteristic of drones (white color), represented on the frequency spectrum (bottom right).

Using the application in Figure 3 we have tested the beamforming capabilities of the system and also directivity, using the spiral microphone array. In this simulation, the atmospheric conditions (turbulence) that may affect the propagation of sounds were not taken into account. Employing a set of multiple microphones with beamforming and a signal processing technique used filtering in order to obtain a better signal reception increased the maximum detection distance in the presented mode.

### 3.2. Extracting the Features of the UAV-Generated Acoustic Signal

**Figure 4:**
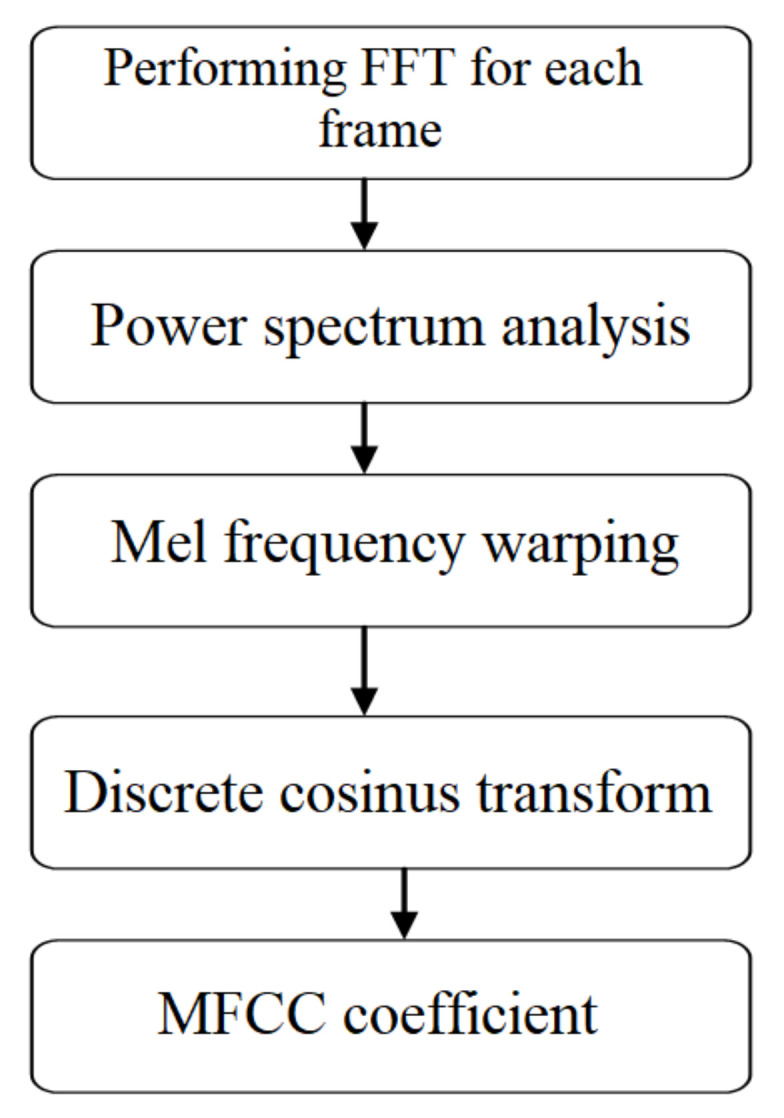
UAV-associated MFCC coefficients extraction procedure.

The process that is common for all forms of acoustic signals recognition systems is the extraction of characteristic vectors from uniformly distributed segments of time of the sampled sound signal. Prior to extraction of these features, the UAV generated signal must undergo the following processes:

(a) Filtering: The detector’s input sound needs filtering to get rid of unwanted frequencies. On the other hand, the filter must not affect the reflection coefficients. In the experiments an IIR notch adaptive filter has been used.
(b) Segmentation: the acoustic signal is non-stationary for a long-time observation, but quasi-stationary for short time periods, i.e., 10–30 ms, therefore the acoustic signal is divided into fixed-length segments, called frames. For this particular case, the size of a frame is 20 ms, with a generation period of 10 ms, so that a 15 ms overlap occurs from one window to the next one.
(c) Attenuation: Each frame is multiplied by a window function, usually Hamming, to mitigate the effect of finishing windows segmentation.
(d) Mel Frequency Cepstrum Coefficients (MFCC) parameters: To recognize an acoustic pattern generated by the UAV, it is important to extract specific features from each frame. Many such features have been investigated, such as linear prediction coefficients (LPCs), which are derived directly from the speech production process, as well as the perceptual linear prediction (PLP) coefficients that are based on the auditory system. However, in the last two decades, spectrum-based characteristics have become popular especially because they come directly from the Fourier transform. The Spectrum-Based Mel Frequency Cepstrum coefficients are employed in this research and their success is due to a filter bank which make use of wavelet transforms for processing the Fourier Transform, with a perceptual scale similar to the human auditory system. Also, these coefficients are robust to noise and flexible, due to the cepstrum processing. With the help of the UAV sonic generated specific MFCC coefficients, recognition dictionaries for the training of neural networks are then shaped.
(e) Feature Extraction for MFCC. The extraction algorithms of the MFCC parameters are shown in Figure 4. The calculation steps are the following:
  - Performing FFT for each frame of the utterance and removing half of it.
  - The spectrum of each frame is warped onto the Mel scale and thus Mel spectral coefficients are obtained.
  - Discrete cosine transform is performed on Mel spectral coefficients of each frame, hence obtaining MFCC.
  - The first two coefficients of the obtained MFCC are removed as they varied significantly between different utterances of the same word.
  - Liftering is done by replacing all MFCC except the first 14 by zero.
  - The first coefficient of MFCC of each frame is replaced by the log energy of the correspondent frame.
  - Delta and acceleration coefficients are found from the MFCC to increase the dimension of the feature vector of the frames, thereby increasing the accuracy.
  - Delta cepstral coefficients add dynamic information to the static cepstral features. For a short-time sequence *C*[*n*], the delta-cepstral features are typically defined as:
    (1) $$D\left[n\right]=C\left[n+m\right]-C\left[n-m\right]$$
    where *n* is the index of the analysis frame and in practice *m* is approximately 2 or 3.
  - Coefficients describing acceleration are found by replacing the MFCC in the above equation by delta coefficients.
  - Feature vector is normalized by subtracting their mean from each element.
  - Thus, each MFCC acoustic frame is transformed into a characteristic vector with size 35 and used to make learning dictionaries for feature training of concurrent neural networks (feature matching).
  A set of 30 MFCC coefficient matrices was created for each drone, corresponding to drone flying the distances (0 to 25 m), (25 to 50 m), (50 to 100 m), (100 to 200 m) and (200 to 500 m).
(f) *The Adaptive Filters.* The role of the adaptive filter is to best approximate the value of a signal at a given moment, based on a finite number of previous values. The linear prediction method allows very good estimates of signal parameters, as well as the possibility to obtain relatively high computing speeds. Predictor analysis is since a sample that can be approximated as a linear combination of the previous samples. By minimizing the sum of square differences on a finite interval, between real signal samples and those obtained by linear prediction, a single set of coefficients called prediction coefficients can be determined. The estimation of model parameters according to this principle leads to a set of linear equations, which can be solved efficiently for obtaining the prediction coefficients.
  Equations (2) and (3) are considered:
  (2) $$x\left(n\right)=\overset{P}{\underset{k=1}{\sum }}a_{k}x\left(n-k\right)+Gs\left(n\right)$$
  (3) $$H\left(z\right)=\frac{G}{1-GA\left(z\right)}$$
  where *H*(*z*) is the acoustic environment feedback, *z* is transfer function of a linear model and $A\left(z\right)=1-\sum_{k=1}^{P}\alpha_{k}z^{-k}$ is the *z* transfer function model of reverberations and multipath reflection of environment, it is noted that it is possible to establish a connection between the gain factor constant, G, the excitation signal and the prediction error. In the case of ak=α=const, the coefficients of the real predictor and of the model are identical: $e\left(n\right)=Gs\left(n\right)$.
  This means that the input signal is proportional to the error signal. Practically, it is assumed that the error signal energy is equal to that of the input signal:
  (4) $$G^{2}\overset{N-1}{\underset{m=0}{\sum }}s^{2}\left(m\right)=\overset{N-1}{\underset{m=0}{\sum }}e^{2}\left(m\right)=\bar{e_{n}}$$
  It should be noted, however, that for the UAV-specific audio signal if $s\left(n\right)=\delta \left(n\right)$, it is necessary for the *p*-order of the predictor to be enough large so as to consider all the effects, eventually the occurrence of the transient waves. In the case of sounds without a specific UAV source, the signal *s*(*n*) is assumed to be white Gaussian noise with unitary variation and zero mean.
(g) Time—Frequency Analysis. The analysis of the acoustic signals can be performed by one-dimensional or two-dimensional methods. One-dimensional methods involve that the analysis is made only in the time domain or only in the frequency domain and generally have low degree of complexity.

Although they have the advantage of offering, in many cases, a way of quickly first evaluating and analyzing signals, in many situations, especially in the case of analyzing the transient values that appear in the acoustic signals generated by the drones, the information that is obtained, regarding the shape and the parameters they is limited and with a low degree of approximation.

The second category of methods, meaning the two-dimensional representations in the time-frequency domain, represent powerful signal analysis tools and it is therefore advisable to use, if the situation allows, a pre-processing of signals, in order to identify transient waves. These representations have the advantage of allowing to emphasize certain “hidden” properties of the signals. From the point of view of the acoustic systems for detecting and analyzing the sound signals generated by the drones, it is of interest to analyze the signals at the lowest level, compared to the noise of the device. Therefore time-frequency analyzes should be performed on signals affected by noise, the signal-to-noise ratio being of particular importance in assessing transient waves. A comparison is shown below in Table 1.

Table 1 compares the properties verified by several time-frequency representations in Cohen’s class. The Cohen class method involves the selection of the nearest nucleus function that corresponds to the fundamental waveform that describes the acoustic signatures specific to drones. Thus, the shape of the nucleus based on the peak values (localization), and the amplitude of a “control” function must be chosen. The frequency resolution corresponding to spectrum analysis, that varies over time, is equal to the Nyquist frequency divided by 2*^n^* (*n* = 8). The resolution in the time domain is 2*^n^* ms (*n* = 4), as required by the applied method.

The class of time-frequency representations, in the most general form has been described by Cohen:

(5) $$C\left(t,\omega ,\Phi \right)=\frac{1}{2\cdot \pi }\int_{-\infty }^{\infty }\exp[j\cdot \left(\xi \cdot t-\tau \cdot \omega -\xi \cdot u\right)]\cdot \Phi (\xi ,\tau )\cdot f\left(u+\frac{\tau }{2}\right)\cdot f^{*}\left(u-\frac{\tau }{2}\right)dud\tau d\xi$$

where *Φ* is an arbitrary function called kernel function. After the choice of this function, several specific cases are obtained corresponding to certain distributions (*t*, *ω*).

The time-frequency representations in Cohen’s class must fulfill certain properties. Compliance with these properties is materialized by imposing certain conditions on the nucleus function. The first two properties relate to the temporal and frequency gap (compatibility with filtering and modulation operations) as follows:

(6) $$P_{1}:\;C_{f_{t_{0}}f}\left(t,\omega ,\Phi \right)=C_{f}\left(t-t_{0},\omega ,\Phi \right)$$

(7) $$P_{2}:\;C_{M_{\omega_{0}}f}\left(t,\omega ,\Phi \right)=C_{f}\left(t,\omega -\omega_{0},\Phi \right)$$

For these conditions to be met, it may be observed that the kernel function Φ must be independent of *t* and ω:

(8) $$C_{1}:\;\Phi \left(\xi ,\tau ,t,\omega \right)\;\mathrm{must}\;\mathrm{be}\;\mathrm{independent}\;\mathrm{of}\;t,$$

(9) $$C_{2}:\;\Phi \left(\xi ,\tau ,t,\omega \right)\;\mathrm{must}\;\mathrm{be}\;\mathrm{independent}\;\mathrm{of}\;\omega .$$

Two other properties that must characterize time-frequency representations refer to the conservation of marginal laws:

(10) $$P_{3}:\;\frac{1}{2\pi }\int_{-\infty }^{\infty }C_{f}\left(t,\omega ,\Phi \right)d\omega ={\left|f\left(t\right)\right|}^{2}$$

(11) $$P_{4}:\;\int_{-\infty }^{\infty }C_{f}\left(t,\omega ,\Phi \right)dt={\left|F\left(\omega \right)\right|}^{2}$$

The restrictions corresponding to these properties that the function must fulfill are:

(12) $$C_{3}:\;\Phi \left(\xi ,\tau \right)=1,\;\forall \xi ;$$

(13) $$C_{4}:\;\Phi \left(0,\tau \right)=1,\;\forall \tau .$$

The function *Φ* must therefore take the following form:

(14) $$\Phi_{\alpha }\left(\xi ,\tau \right)=\exp\left(j\cdot \alpha \cdot \xi \cdot \tau \right)$$

For time-frequency representations to be real, the following condition is to be met:

(15) $$P_{5}:\;C_{f}\left(t,\omega ,\Phi \right)=C_{f}^{*}\left(t,\omega ,\Phi \right)$$

This happens only if:

(16) $$C_{5}:\;\Phi \left(\xi ,\tau \right)=\Phi \left(-\xi ,-\tau \right).$$

The most representative time-frequency distributions in Cohen’s class are presented in Table 2.

According to Table 1 and Table 2, it becomes easy to note that the Wigner-Ville transform has the highest number of properties, which justifies the special attention that will be given hereafter.

The Wigner-Ville Distribution. The Wigner-Ville interdependence of two signals is defined by:

(17) $$W_{f,g}\left(t,\omega \right)=\int_{-\infty }^{\infty }f\left(t+\frac{\tau }{2}\right)\cdot g^{*}\left(t-\frac{\tau }{2}\right)\cdot e^{-j\cdot \omega \cdot \tau }d\tau .$$

The Wigner-Ville self-distribution of a signal is given by:

(18) $$W_{f}\left(t,\omega \right)=W_{f,f}\left(t,\omega \right)=\int_{-\infty }^{\infty }f\left(t+\frac{\tau }{2}\right)\cdot f^{*}\left(t-\frac{\tau }{2}\right)\cdot e^{-j\cdot \omega \cdot \tau }d\tau .$$

The Wigner-Ville distribution can be regarded as a short Fourier transform in which the window continuously adapts with the signal because this window is nothing but the signal itself, reversed over time. The Wigner-Ville transform is thus obtained as a result of the following operations:

(a) at any moment *t*, multiply the signal with the conjugate “mirror image”, relative to the moment of evaluation:
  (19) $$q_{x}\left(t,\tau \right)=f\left(t+\frac{\tau }{2}\right)\cdot f^{*}\left(t-\frac{\tau }{2}\right)$$
(b) calculate the Fourier transform for the result of this multiplication, in relation to the offset variable τ.

One of the properties of this time-frequency representation is that it can also be defined starting from the spectral functions:

(20) $$W_{F,G}\left(\omega ,t\right)=\frac{1}{2\pi }\int_{-\infty }^{\infty }F\left(\omega +\frac{\xi }{2}\right)\cdot F^{*}\left(\omega -\frac{\xi }{2}\right)\cdot e^{j\cdot \xi \cdot t}d\xi$$

It is thus obtained:

(21) $$W_{F,G}\left(\omega ,t\right)=W_{f,g}\left(t,\omega \right).$$

### 3.3. Analysis of UAVs Specific Acoustic Signals Employing Cohen (Wigner-Ville) Energy Distributions

**Figure 5:**
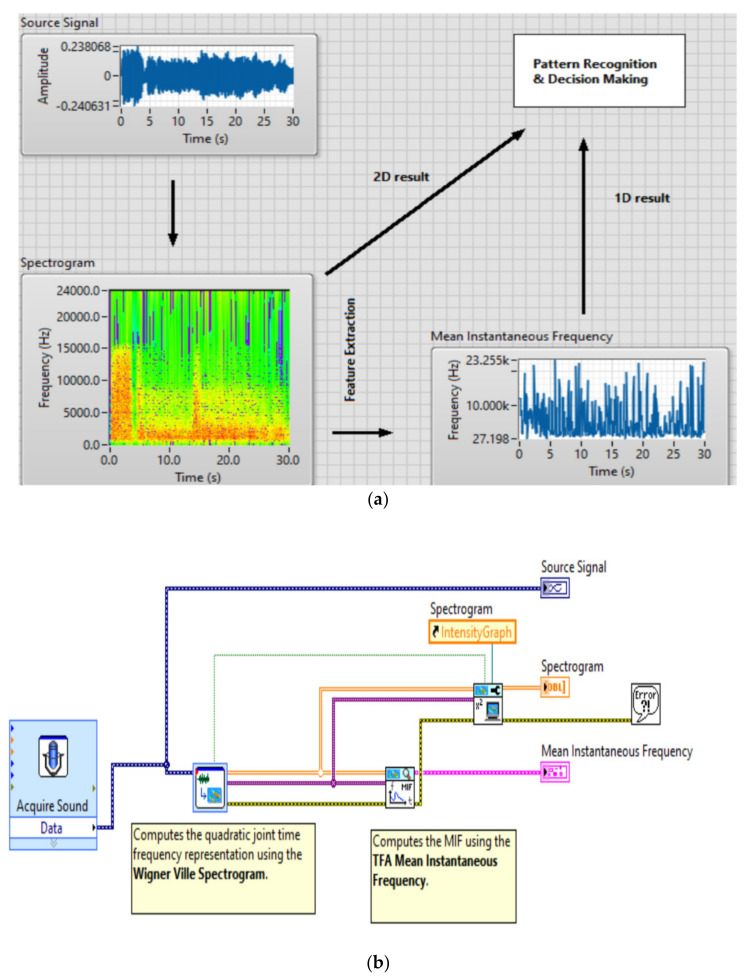
The Wigner-Ville analysis performed for an acoustic signal generated by an UAV, GUI interfaces (**a**) and software implementation (**b**).

Using the application presented in Figure 5, the spectrograms related to the sounds produced by UAVs are obtained, and the results are used to the neuronal network training files. For training 30 files with Wigner-Ville spectrograms were made, each file having 200 spectrograms images of 128 × 128 dimension. In total a few 6000 training spectrograms for neuronal network have been employed.

The presented quadratic representations, which are part of the broader category described by Cohen’s class, provide excellent time-frequency analysis properties of acoustic signals. Following the carried out experiments, some important aspects can be emphasized regarding the use of the analysis of the acoustic signals generated by the drones using the Wigner-Ville time-frequency distributions, of Cohen’s class, namely:

- The energy structure of the analyzed signals can be identified and located with a good accuracy in the time-frequency plane.
- When the type, duration, frequency, and temporal arrangement of the signals are not a priori known, they can be estimated using time-frequency distributions.
- The possibility of implementing these analysis algorithms in systems for analyzing the transient acoustic signals generated by the drones becomes thus available.

Useful databases can be created to identify the transient acoustic signals generated by the drones detected in the environment, as their “signature” can be individualized using the Wigner-Ville time-frequency representations.

## 4. Employing Concurrent Neural Networks in UAVs Classification Process

### 4.1. Artificial Neural Networks and Wigner-Ville Spectrograms, MFCC, Mean Instantaneous Frequency (MIF) Classes

This algorithm implements the concept of competition at the level of a collection of neural networks and determines the importance of the inputs which influence the performances in the recognition of the acoustic fingerprint, using neural networks. It is known that modularity and the “divide et impera” principle applied to neural networks can improve their performance [27].

The algorithm employs the model of concurrent neural networks (CoNN) that combines the paradigms of supervised and unsupervised learning and offers an optimal solution for detecting acoustic fingerprints specific to UAVs. The principle of competition is used in this process within a collection of neural networks that are independently trained to solve different subproblems.

The CoNN training was performed offline using the system in Figure 1, and the training data was performed for 3 available drones, corresponding different flying distances (0 to 25 m), (25 to 50 m), (50 to 100 m), (100 to 200 m) and (200 to 500 m).

There have been tested three quadcopter models: a DJI Phantom 2 (mini class), DJI Matrix 600 (medium class) and a homemade drone (medium class).

The first training data were gathered in an anechoic chamber, for the drones specified in the article at different engine speeds, sampling frequency 44 kHz. The 2nd training data set: the drone sound data was recorded in a quiet outdoor place (real-life environment without the polyphonic sound environment typical of outside areas, such as on the rooftop of a building in a calm place or isolated environment) at successive distances of 20, 60 and 120 m for two types of behavior, hovering and approaching, with a total time of 64 s. Exact labeling of the drone sound was achieved by starting to record after the drone is activated and stopping before deactivation.

Recognition is performed by identifying the neural network that provides the best response. The experiments performed demonstrated that, compared to the cases where competition is not used at all, the obtained recognition process accuracy was higher when employing the model proposed in the present solution. The system consists of neural networks with various architectures. Multilayer perceptron type modules were employed, along with time delay neural network and self-organizing map types. The recognition scheme consists of a collection of modules trained on a subproblem and a module that selects the best answer. The training and recognition algorithms, presented in Figure 4 and Figure 5, implement these two techniques for multilayer perceptrons, time-delayed neural networks and self-organizing maps. Multi-layer perceptron—concurrent neural network (MLP-CoNN) and time-delay neural network—concurrent neural network (TDNN-CoNN) use supervised trained modules and the training vector sets contain both positive and negative examples. In contrast, self-organizing maps—concurrent neural network (SOM-CoNN) consists of modules that are trained by an unsupervised algorithm and the data consist of only positive examples.

Modular/competing neural networks are based on the idea that, in addition to the hierarchical levels of organization of artificial neural networks: synapses, neurons, neuron layers and the network itself, a new level can be created by combining several neural networks. The model proposed in this article, called concurrent neural networks, introduces a neural recognition technique that is based on the idea of competition between multiple modular neural networks which work in parallel using the NI board controller [27,28]. The number of networks used is equal to the number of classes in which the vectors are grouped, and the training is supervised. Each network is designed to correctly recognize vectors in a single class, so that the best answers appear only when vectors from the class with which they were trained are presented. This model is in fact a framework that offers architecture flexibility because the modules can be represented by different types of neural networks.

Starting from the CoNN model proposed in this work, the concurrent self-organizing maps (CSOM) model has been introduced, which detaches itself as a technique with excellent performance to implementation on FPGA and Intel Core i7 processor (3.1 GHz).

**Figure 6:**
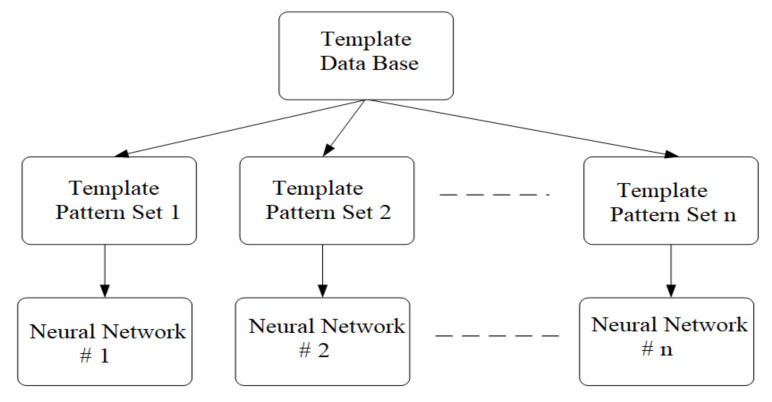
Scheme used to train competing neural networks.

The general scheme used to train competing neural networks is presented in Figure 6. In this scheme, *n* represents the number of neural networks working in parallel, but it is also equal to the number of classes in which the training vectors are grouped [29,30].

The *X* set of vectors is obtained from the preprocessing of the acquired audio signals for the purpose of network training. From this set are extracted the sets of vectors *Xj*, *j =* *1, 2 … n* with which the *n* neural networks will be trained. Following the learning procedure, each neural network will have to respond positively to a single class of vectors and to give negative responses to all other vectors. The training algorithm for the competing network is as follows:

Step 1. Create the database containing the training vectors obtained from the preprocessing of the acoustic signal.
Step 2. The sets of vectors specific to each neural network are extracted from the database. If necessary, the desired outputs are set.
Step 3. Apply the training algorithm to each neural network using the vector sets created in Step 2.

**Figure 7:**
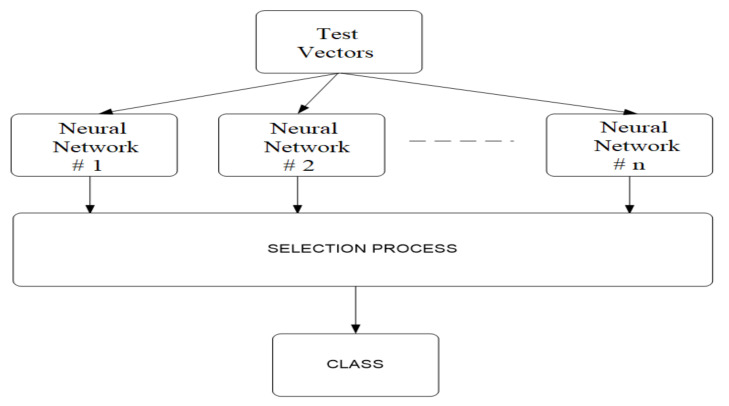
Diagram for UAV recognition and classification using CoNN.

Recognition and classification using CoNN, is performed in parallel, using the principle of competition, according to the diagram in Figure 7. It is assumed that the neural networks were trained by the algorithm described above. When applying the test vector, the networks generate an individual response, and the selection consists of choosing the network that generated the strongest response. The network selected by the winner rule is declared winner. The index of the winning network will be the index of the class in which the test vector is placed. This method of recognizing features therefore implies that the number of classes with which the competing network will work is a priori known and that there are sufficient training vectors for each class. The recognition algorithm is presented in the following steps:

Step 1. The test vector is created by preprocessing the acoustic signal.
Step 2. The test vector is transmitted in parallel to all the previously trained neural networks.
Step 3. The selection block sets the network index with the best answer. This will be the index of the class in which the vector is framed.

The models can be customized by placing different architectures instead of the neural networks. Multilayer perceptron (MLP), time delay neural networks (TDNN) and Kohonen (SOM) maps were used for this work, thus obtaining three different types of competing neural networks.

### 4.2. Multiple Drone Detection and Confusion Matrix

This section deals with the experiments performed on the problem of multiple drone detection with the custom collected dataset. The experiments are organized in the following order:

(1) Concurrent Neural Networks (CoNN) with Wigner-Ville spectrogram class.
(2) Concurrent Neural Networks (CoNN) with MFCC dictionary class.
(3) Concurrent Neural Networks (CoNN) with MIF class.

#### 4.2.1. Confusion Matrix

To establish the performance values it is necessary to calculate the confusion matrix. The confusion matrix consists of real values on one dimension and predicted labels for the second dimension, and each class consists of a row and a column. The diagonal elements of the matrix represent the correctly classified results.

The values calculated from the confusion matrix represent Precision, Recall and F1-score which is the harmonic average of the accuracy and recall and the accuracy of the classification:

Precision: it is defined as the number of samples which contain the existence of the drone.

Recall: It is defined that the ratio between the expected number of samples to contain a drone and the number of samples that contain the drone.

F-measure: It is defined as the harmonic average between accuracy and recall.

F1 scores are calculated for each class, followed by the average of the scaled scores with weights. The weights are generated from the number of samples corresponding to each class.

#### 4.2.2. CoNN with Wigner-Ville Spectrograms

The spectrograms extracted in the experiment are transformed into logarithmic domain. The transformed features are used as the input to the model. The network is trained for 200 epochs with batch size of 16. This experiment resulted in classification accuracy of 91 percent, Recall is 0.96, and microaverage F1-score is 0.91. The confusion matrix is shown in Table 3 and classification report is show in Table 4.

#### 4.2.3. CoNN with MFCC

For the MFCC dictionary extracted in the experiment, the Mel filter bank with 128 Mel filters is applied. CoNN is trained for 200 epochs with batch size of 128 sample. This experiment resulted in classification accuracy of 87 percent, Recall is 0.95, and micro-average F1-score is 0.86. The confusion matrix is shown in Table 5 and classification report is show in Table 6.

#### 4.2.4. CoNN with MIF

CoNN is trained for 200 epochs with batch size of 16. This experiment resulted in classification accuracy of 85.3 percent, Recall is 0.95, and micro-average F1-score is 0.86. The confusion matrix is shown in Table 7 and classification report is show in Table 8.

## 5. Experimental Results

After observing that the CoNN model shows remarkable improvements of recognition rates of acoustic fingerprints compared to the classic models, this section will focus on the recognition and identification of UAVs’ specific acoustic signals. A training database was created using the Wigner-Ville spectrogram, MFCC and MIF dictionaries corresponding to the acoustic signals of 6 multirotor drones. We tested six multirotor models: (1) DJI Matrice 600 (medium), (2–4) homemade drones (medium and large) three units, (5) DJI Phantom 4 (mini), (6) Parrot AR drone 2 (mini).

The drone was tracked outdoors on a test field between buildings, a street with pedestrian and cars/tram traffic nearby (urban conditions). The atmospheric conditions for the real-time tests were sunny weather, temperature 30–35 degrees Celsius, precipitation 5%, humidity 73%, wind 5 km/h, atmospheric pressure 1013 hPa (101.3 kPa) and presence of noise in urban conditions (source: National Agency for the weather).

Each of these drones were tested ten times. For each iteration, the training vectors of the recognition system were extracted from the first five data sets, keeping the next five data sets for testing. In this way, 200 training sets were obtained for the preparation of the system and another two hundred for its verification. In addition to speaker recognition, a set of experiments was performed using a first unique neural network to recognize the model and then CoNN.

The results obtained in the real-time tests are presented in Figure 8, Figure 9, Figure 10, Figure 11, Figure 12, Figure 13, Figure 14, Figure 15, Figure 16, Figure 17 and Figure 18.

⮚ Model DJI PHANTOM 4, type of classification—small (5)

**Figure 8:**
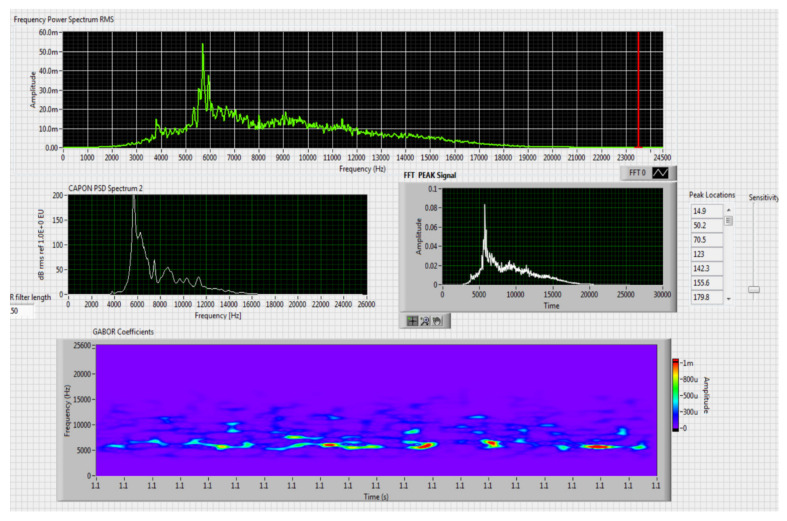
Results obtained for stationary distance 50 m, altitude 3 m.

For this stage only the Kohonen network was tested, given the results that were obtained in recognition speakers and their behavior compared to that of a CoNN. For the variant that uses a single SOM, the network was trained with the whole sequence of vectors obtained after preprocessing the selected acoustic signals. A Kohonen network was trained in two stages with 10 × 15 nodes through the self-organizing feature map (SOFM) algorithm.

The first stage, the organization of clusters, took place along 1000 steps and the neighborhood gradually declined to a single neuron. In the second stage, the training was performed in 10,000 steps and the neighborhood remained fixed to the minimum size. Following training and calibration of the neural network with the training vectors we obtained a set of labeled (annotation) prototypes whose structure is that of Table 6. The applied technique for recognition is described below. The acoustic frequencies identified in the test signal are preprocessed by means of a window from which a vector of the component parts is calculated.

**Figure 9:**
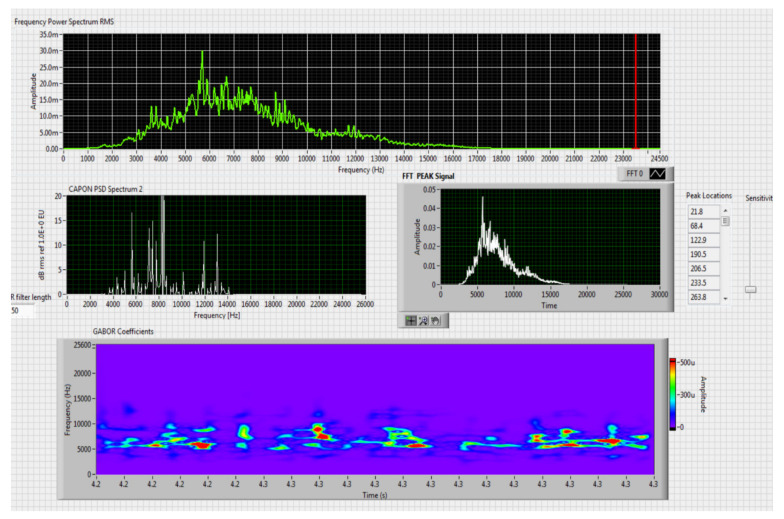
Results obtained for stationary distance 150 m, altitude 3 m.

⮚ Model Homemade multirotor, type of classification—medium (2)
  **Figure 10:**
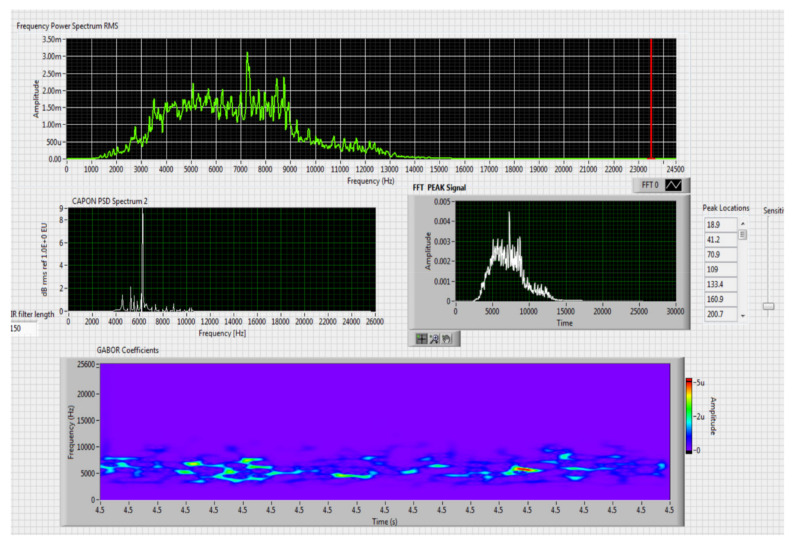
Results obtained for moving distance 50–250 m, altitude 4 m.

**Figure 11:**
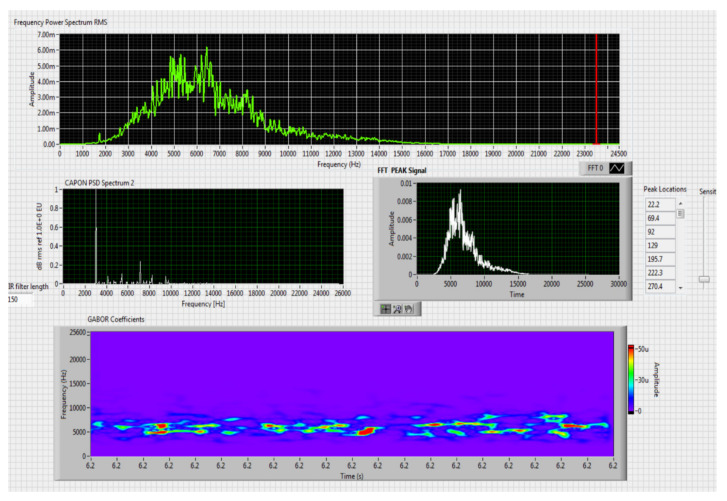
Results obtained for stationary distance 150 m, altitude 4 m.
⮚ Model DJI Matrice 600, type of classification—medium (1)
  **Figure 12:**
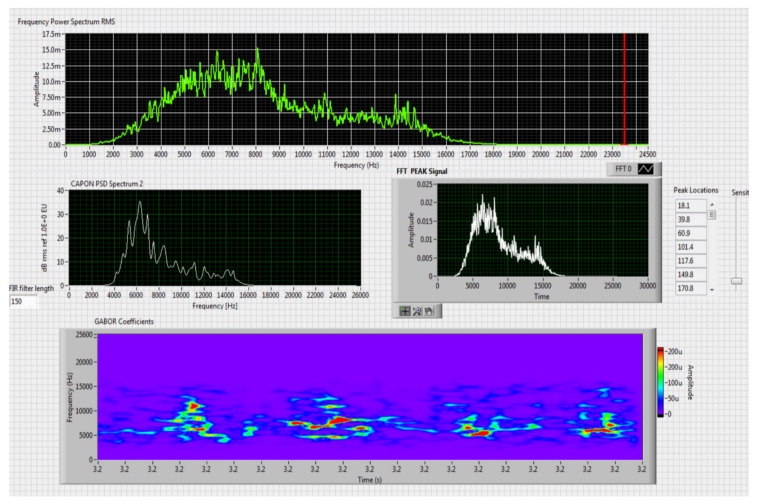
Results obtained for stationary distance 150 m, altitude 4 m.

**Figure 13:**
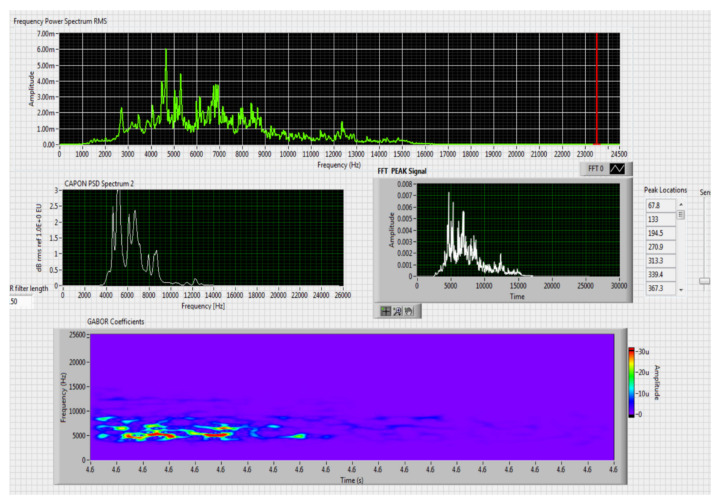
Results obtained for stationary distance 150 m, altitude 10 m.
⮚ Model Homemade Octocopter, type of classification—medium (3)
  **Figure 14:**
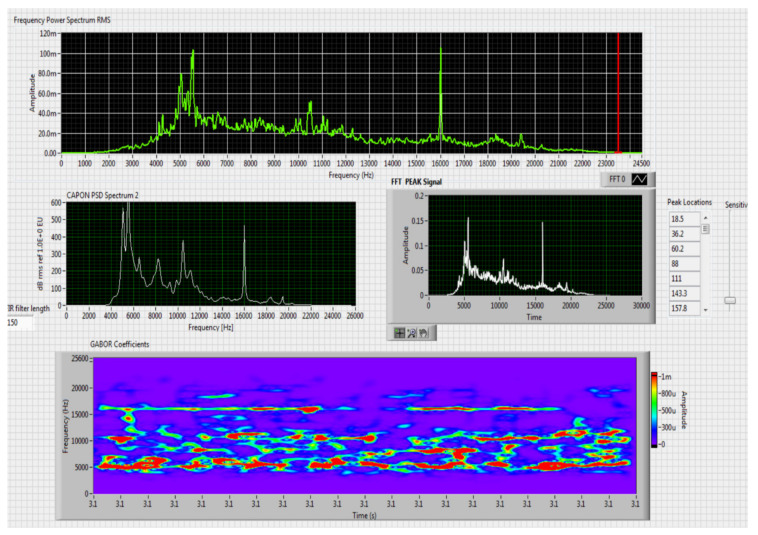
Results obtained for stationary distance 150 m, altitude 3 m.

**Figure 15:**
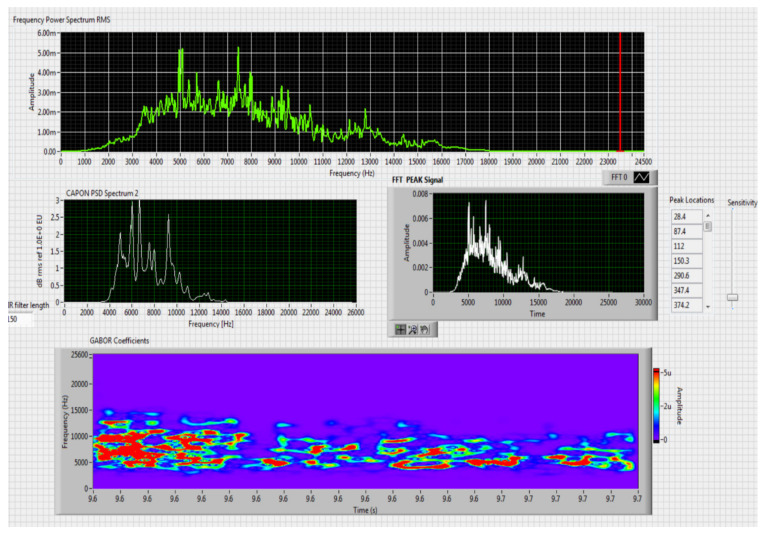
Results obtained for stationary distance 380 m, altitude 3 m.
⮚ Model Homemade Octocopter, type of classification—large (4)
  **Figure 16:**
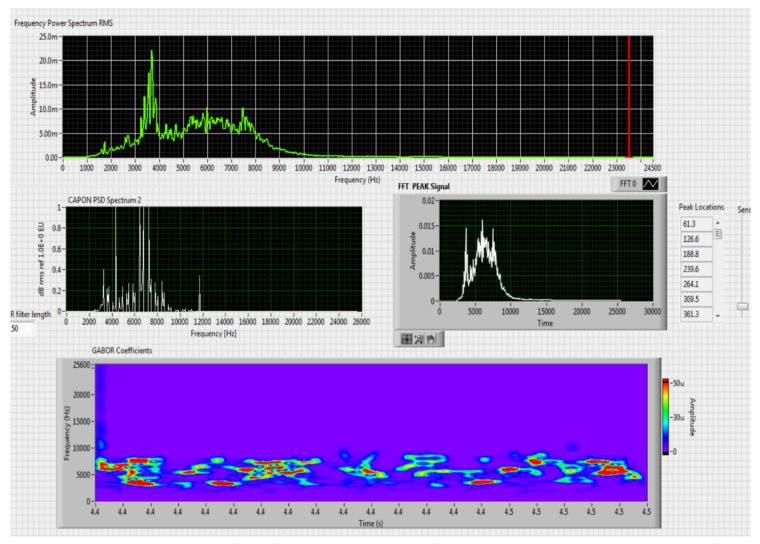
Results obtained for moving distance 100–380 m, altitude 60 m.
⮚ Parrot AR drone 2 (mini), type of classification—small (6)
  **Figure 17:**
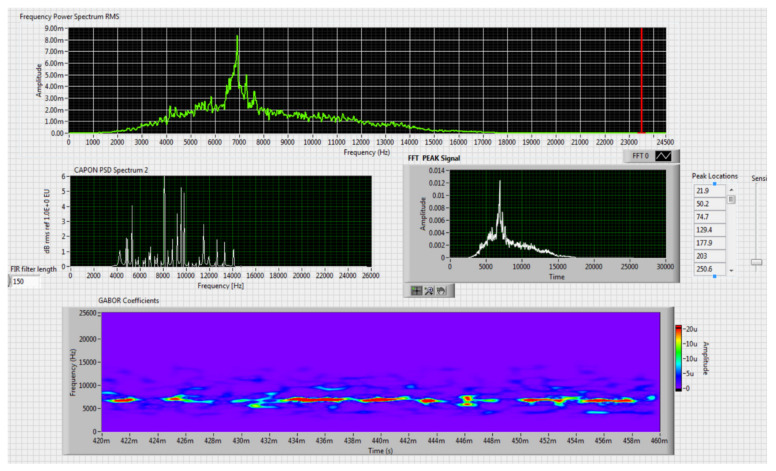
Results obtained for stationary distance 130 m, altitude 2 m.

**Figure 18:**
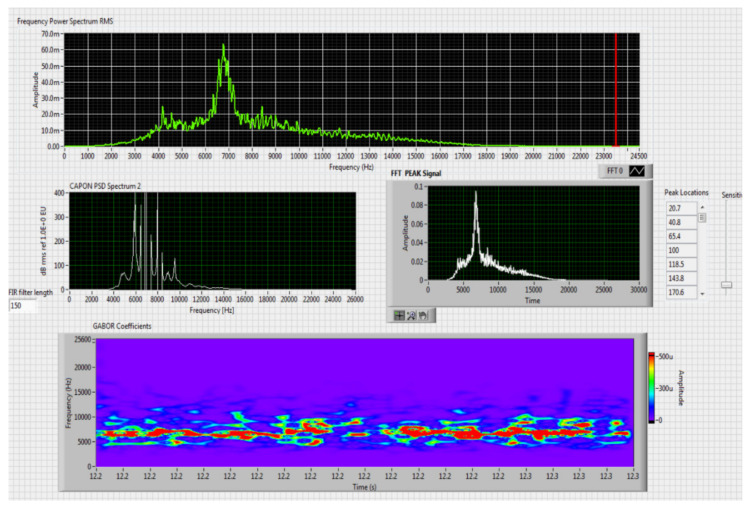
Results obtained for stationary distance 80 m, moving altitude 2–10 m.

The description of the technique applied for recognition continues. The frequencies identified in the test signal are preprocessed by the means of a window from which a vector of the component parts is calculated. The window moves with a step of 50 samples and a collection of vectors is obtained, whose sequence describes the evolution of the acoustic signal specific to the drones. For each vector, the position that corresponds to the signal is kept, and the minimum quantization error, i.e., the tag that the neural network calculates. Experimentally, a maximum threshold for quantization error was set to eliminate frequencies that are supposed not belonging to any class. Through this process, a sequence of class labels that show how the acoustic signals specific to the drones were recognized by the system was obtained.

In Table 9 the experimental results are presented in percentages of recognition and identification of the drones with SOM and CoNN.

In Table 10, when we refer to the “Accuracy” of “CoNN”, we refer to a different top-level architecture that:

(1) Takes raw audio data and creates functions for each of the three mentioned networks
(2) Run the data through each network and get an “answer” (a distribution of the probability of class predictions)
(3) Select the “correct” output of the network with the highest response (highest class confidence)
(4) This architecture being explained in Figure 7.

The general classifier based on Concurrent Neural Networks, providing the same test framework for all 30 training files has been tested. Using the maximum win strategy, the output tag was identified, with a resulting precision of the drone recognition is 96.3%.

### 5.1. Computational Time

The time required to extract the characteristics of a 256 × 250 spectrogram image using CoNN is 1.26 s, while the time required to extract the characteristics of an MFCC and MIF sample from audio samples is 0.5 s. The total training time required the model for the spectrograms image data set was 18 min, while the model training time for the MFCC and MIF audio sample are 2.5 min. The time required to train the combined model data set was 40 min. The trained model classifies objects in 3 s.

### 5.2. Comparison with Other Similar Methods

Comparing the method proposed in this article with similar methods presented in the literature for drone detection using acoustic signature, which uses a supervised learning machine, the authors report detection accuracies between 79% and 98.5%, without mentioning the detection distance of the signals acoustics generated by drones [31,32,33,34,35]. The method proposed by us has an average accuracy of almost 96.3% for detecting the sounds generated by the drone, for a distance between 150 m for small class drones and 500 m for middle and large class drones. Our tests were performed in a test range with a maximum length of 380 m, but from the results shown in Figure 8, Figure 9, Figure 10, Figure 11, Figure 12, Figure 13, Figure 14, Figure 15, Figure 16, Figure 17 and Figure 18, it results that the detection distance of the acoustic signals from the drones reaches approximately 500 m, for different classes of drones. The proposed CoNN model classifies objects in about 4 s, this time being sufficient for warning because the network of microphone areas is distributed in width and depth, thus creating a safety zone.

## 6. Discussion and Conclusions

This paper investigates the effectiveness of machine learning techniques in addressing the problem of UAV unauthorized flight detection, in the context of critical areas protection. For extracting the acoustic fingerprint of an UAV, a time-frequency analysis using Wigner-Ville is adopted by the warning system, to recognize specific acoustic signals. Dictionaries MFCC and MIF (Mean Instantaneous Frequency) coefficients specific to each type of drone have been also added in this process to improve the recognition precision of CoNN.

The contributions of the proposed solution are the following:

- Development of a spiral microphone array, combining microphones in the audible and ultrasonic fields, set in an interchangeable configuration with multichannel adaptive weights.
- introduction of the possibility of detecting low intensity acoustic signals specific to multirotor mini drones, at a distance of ~120 m.
- Dhe development of the training base with the Wigner-Ville spectrogram, MFCC dictionaries and MIF coefficients.
- The use of multilayer perceptron modules, time delay neural networks and self-organizing maps.
- The use of a set of networks equal to the number of classes in which the vectors are grouped, with a supervised preparation.
- The recognition scheme consists of a collection of models trained on a subproblem and a module that selects the best answer.
- Tests have shown that large multirotor (diameter 1.5 m) can be detected at a distance of ~500 m, and medium multirotor (diameter less than 1 m) can be detected at a distance of at least 380 m.
- The possibility of integrating the microphone area in a network structure (scalability), which can be controlled by a single cRIO system by integrating several acquisition boards. The placement of the acoustic sensors within the network can be done linearly and in depth, so that a safety zone can be created around the perimeter restricted for the flight of drones.

From the results obtained in the experiments performed, engineered features employed on CoNN proved to have better performances. CoNN architectures have resulted in better generalization performance and faster convergence for spectro-temporal data. The Wigner-Ville spectrograms show improved performance among other spectrogram variants (for example transformed into short-term FFT–STFT).

The results obtained with both the datasets lead to the conclusion that multiple drone detection employing audio analysis is possible. In future work, as presented in [36], a video camera for drone detection and recognition will be integrated in the microphone area. The two modules, acoustic and video, will work in parallel and the results will be integrated to increase the recognition capacity and classification of drones. A final radio detection (RF) module will also be integrated on the final architecture, and the results will be displayed in a command and control system.

## 7. Patents

Part of this research has been previously tested for developing a method for anonymous collection of travelers flowing in a public transport system and resulted in a patent application: RO A/00493, “Method and System for Anonymous Collection of Information regarding Position and Mobility in Public Transportation, employing Bluetooth and Artificial Intelligence” in 2019.

Results of this research culminated in a patent application: RO A/00331, “System and Method for Detecting Active Aircraft (Drone) Vehicle by Deep Learning Analysis of Sound and Capture Images” in 2020.

## Figures and Tables

**Table 1:** Properties verified by several time-frequency representations in Cohen’s class.

Property	Spectrogram	Wigner-Ville	Born-Jordan	Choi-Williams
Energy	√	√	√	√
Time limitation	-	√	√	√
Frequency limitation	-	-	-	
Reality	√	√	√	√
Positive Value	√	-	-	-
Causality	-	-	-	-
Reversibility	-	√	-	√
Expansion	-	√	√	√
Filtering	-	√	-	-
Modularity	√	√		
Temporal support	-	√	√	-
Frequency support	-	√	√	-
Unit value	-	√	-	-
Instantaneous frequency	-	√	√	√
Group delay	-	√	√	√

**Table 2:** Time-frequency representations of Cohen’s class and associated kernel functions.

$\Phi \left(\xi ,\tau \right)$	$C_{x}\left(t,\nu ,\Phi \right)$	Distribution Type
1	$\int_{-\infty }^{\infty }f\left(t+\frac{\tau }{2}\right)\cdot f^{*}\left(t-\frac{\tau }{2}\right)\cdot e^{-i\cdot 2\pi \cdot \nu \cdot \tau }d\tau$	Wigner-Ville
$\frac{sin\left(\pi \xi \tau \right)}{\pi \xi \tau }$	$\int_{-\infty }^{\infty }\left[\frac{1}{\left|r\right|}\int_{t-\frac{\tau }{2}}^{t+\frac{\tau }{2}}f\left(s+\frac{\tau }{2}\right)\cdot f^{*}\left(s-\frac{\tau }{2}\right)ds\right]\cdot e^{-i\cdot 2\pi \cdot \nu \cdot \tau }d\tau$	Born-Jordan
$e^{\frac{-{\left(\pi \xi \frac{\tau }{\sigma }\right)}^{2}}{2}}$	$\int_{-\infty }^{\infty }\int_{-\infty }^{\infty }\frac{\sigma }{\left|r\right|}\cdot e^{-2\sigma^{2}{\left(s-t\right)}^{2}\cdot \tau^{2}}\cdot f\left(s+\frac{\tau }{2}\right)\cdot f^{*}\left(s-\frac{\tau }{2}\right)e^{-i\cdot 2\pi \cdot \nu \cdot \tau }dsd\tau$	Choi-Williams
$A_{h}^{*}\left(\xi ,\tau \right)$	${\left|\int_{-\infty }^{\infty }f\left(s\right)\cdot h^{*}\left(s-t\right)\cdot e^{-i\cdot 2\pi \cdot \nu \cdot s}ds\right|}^{2}$	Spectogram

**Table 3:** The confusion matrix from CoNN with Wigner-Ville spectrogram classes.

		Predict Label		
		Background Noise	Single Drone	Two Drone
True Label	Background Noise	1.00	0.00	0.00
	Single Drone	0.00	0.94	0.06
	Two Drone	0.00	0.23	0.77

**Table 4:** Classification Report for CoNN with Wigner-Ville spectrogram.

Classes	Precision	Recall	F1—Score
Backgroud Noise	1	1	1
Single Drone	0.80	0.94	0.87
Two Drone	0.93	0.77	0.84
Avg/total	0.91	0.90	0.90

**Table 5:** The confusion matrix from CoNN with MFCC classes.

		Predict Label		
		Background Noise	Single Drone	Two Drone
True Label	Background Noise	0.89	0.08	0.03
	Single Drone	0.03	0.84	0.13
	Two Drone	0.03	0.13	0.85

**Table 6:** Classification Report for CoNN with MFCC.

Classes	Precision	Recall	F1—Score
Backgroud Noise	0.93	0.89	0.91
Single Drone	0.80	0.85	0.82
Two Drone	0.85	0.84	0.84
Avg/total	0.86	0.86	0.86

**Table 7:** The confusion matrix from CoNN with MIF classes.

		Predict Label		
		Background Noise	Single Drone	Two Drone
True Label	Background Noise	0.98	0.01	0.01
	Single Drone	0.09	0.86	0.06
	Two Drone	0.07	0.16	0.77

**Table 8:** Classification Report for CoNN with Mean Instantaneous Frequency.

Classes	Precision	Recall	F1—Score
Backgroud Noise	0.86	0.98	0.91
Single Drone	0.83	0.84	0.83
Two Drone	0.92	0.78	0.84
Avg/total	0.88	0.88	0.88

**Table 9:** Set number of labeled (annotation) prototypes structure has been obtained.

UAV No.	1	2	3	4	5	6
**Training**	472	616	439	625	553	54
**SOM**	45	38	14	25	25	4
**CoNN**	27	27	27	27	27	27

**Table 10:** Experimental results [%] of recognition and identification of the drones.

Drone Model	SOM Accuracy	CoNN Accuracy	Recognition Distance [m]	Drone Class
**1**	73.65%	85,21%	~380	medium
**2**	60.47%	94.95%	~380	medium
**3**	50%	97.34%	~380	medium
**4**	5.89%	90.43%	~500	large
**5**	15.03%	82.37%	~150	small
**6**	45%	85.6%	~150	small
